# Differential Antioxidant Capacities of Human Endometriotic and Endometrial Cell Models Under H_2_O_2_ Exposure

**DOI:** 10.3390/ijms27094131

**Published:** 2026-05-05

**Authors:** Julia A. Coelho, Kaio S. Gomes, Giselle Cerchiaro

**Affiliations:** Metal Biochemistry and Oxidative Stress Laboratory, Centre for Natural Sciences and Humanities, Federal University of ABC, Santo Andre 09280-560, SP, Brazil

**Keywords:** endometriosis, oxidative stress, redox imbalance, N-acetyl cysteine

## Abstract

Endometriosis is associated with oxidative stress and debilitating symptoms, yet its pathophysiology remains incompletely understood, and current treatments are still limited. In this study, oxidative stress responses were compared in 2D and 3D cultures of 12Z and Ishikawa cells using hydrogen peroxide (H_2_O_2_) as a pro-oxidant and N-acetylcysteine (NAC) as an antioxidant. We evaluated H_2_O_2_ sensitivity, Reactive Oxygen Species (ROS) production, glutathione redox homeostasis, and biomolecular damage. The results showed that 12Z cells display greater vulnerability to oxidative stress than Ishikawa cells, with higher basal ROS levels (*p* < 0.01) and increased sensitivity to H_2_O_2_. In 3D culture, 12Z cells exhibited a 72% depletion of total glutathione under oxidative stress, a response not observed in 2D cultures, which instead showed a compensatory pattern. This vulnerability was further supported by increased lipid peroxidation and protein carbonylation. Although NAC restored cell viability and protected lipids and proteins, it did not prevent DNA damage. Together, these findings demonstrate marked differences in antioxidant responses between the two cell models and reinforce the value of 3D systems for investigating oxidative stress-related mechanisms. These results provide mechanistic insights relevant to endometriosis-associated redox imbalance and support further investigation of glutathione dysregulation and ROS-mediated damage in disease-related contexts.

## 1. Introduction

Endometriosis is a chronic inflammatory condition that affects about 10% of women of reproductive age and is characterized by the presence of endometrial-like tissues outside the uterus, usually found in the peritoneum, ovaries and rectovaginal septum [1,2,3,4]. Besides the chronic pelvic pain, about 40% of diagnosed women present infertility, and current treatments focus on symptom management with surgical intervention, through the combination of hormones and laparoscopy, resulting in several side effects and a high rate of recurrence of endometriotic lesions [5,6,7,8].

Since 1920, the main theory regarding the origin of endometriosis has been John A. Sampson’s hypothesis of retrograde menstruation. Over the decades, new hypotheses have emerged, including the lymphovascular dissemination of stem cells, the transdifferentiation of peritoneal coelomic epithelial cells, and embryonic remnants in response to estrogen [9,10,11,12,13,14,15,16]. However, regardless of the proposed theory, all result in activated macrophages and apoptosis of endometrial cells and erythrocytes, leading to the presence of free iron derived from hemoglobin, promoting the cyclic overproduction of H_2_O_2_ and other ROS, and subsequent inflammatory signaling [17].

Clinical studies analyzing fluids and tissues from women diagnosed with endometriosis identified oxidative stress biomarkers, such as 8-hydroxy-2′-deoxyguanosine (8-OHdG) adducts resulting from DNA damage, lipid peroxidation products such as Malondialdehyde (MDA), protein products of advanced oxidation, pro-inflammatory cytokines, and others [18,19,20,21]. Thus, the overproduction of mediators resulting from oxidative stress can be attributed as one of the sources of endometrial dysfunction contributing to disease progression [22].

Given the correlation between disease occurrence and pro-inflammatory markers, it is interesting to study these pathways as targets for alternative therapies [23]. Traditional antioxidants, such as N-acetylcysteine (NAC), can serve as inspiration for such discoveries [24,25,26,27], as it participates in several relevant cellular mechanisms, such as Glutathione (GSH) replenishment, H_2_O_2_ scavenging, activation of the Nuclear Factor E2-related factor 2 (Nrf2) antioxidant defense pathway, prevention of apoptosis by inhibition of Nuclear Factor Kappa-light-chain-enhancer of activated B cells (NF-κB), and increased cytotoxic capacity of immune cells [24,28,29].

Based on that, this work aimed to investigate oxidative stress responses relevant to endometriosis-associated inflammatory and hemolysis-related processes, using H_2_O_2_ exposure as a controlled pro-oxidant stimulus, and to evaluate its effects on biomolecular oxidation, the protective effect of NAC, and the biochemical profiles of two widely used models of immortalized endometrial cell lines—Ishikawa cells, which were immortalized from human endometrial epithelial adenocarcinoma and retain several characteristics of endometrial epithelium, allowing their use in studying normal endometrial behavior and functions [30,31], and the 12Z cell line, immortalized in the 2000s from peritoneal endometrial biopsies, and widely used in constructing in vitro endometriosis models, as it expresses the same gene patterns found in women diagnosed with the disease [32,33,34,35].

The pathogenesis of endometriosis is also deeply driven by genetic predispositions that establish an altered cellular microenvironment [33,36,37]. At the regulatory level, microRNA dysfunction acts as a key genetic determinant by modulating gene expression related to cell survival, proliferation, and immune signaling; their systemic regulation in affected women suggests an epigenetic scenery that favors pro-inflammatory signaling and impaired apoptosis. This susceptibility is further evidenced by a constitutive imbalance in estrogen metabolism enzymes, suggesting a genetically programmed disturbance in metabolic clearing [2]. This enzymatic imbalance leads to the accumulation of reactive hydroxy-estrogens and altered ROS production, acting as a biochemical trigger for the proliferation of ectopic endometrial tissue rather than being a mere byproduct of established lesions.

However, despite extensive evidence linking oxidative stress to endometriosis pathophysiology, comparative studies evaluating the biochemical oxidative responses of cellular models relevant to endometrial and endometriosis-associated biology under controlled oxidative challenge remain limited. Thus, the hypothesis of this work was that, compared to Ishikawa cells, 12Z cells would exhibit greater oxidative vulnerability, including increased sensitivity to H_2_O_2_, elevated production of Reactive Oxygen Species (ROS), depletion of the glutathione antioxidant system, and greater damage to the evaluated biomolecules.

## 2. Results

### 2.1. 12Z Cells Display Markedly Increased Sensitivity to H_2_O_2_ Compared to Ishikawa Cells Across 2D and 3D Models

**Figure 1:**
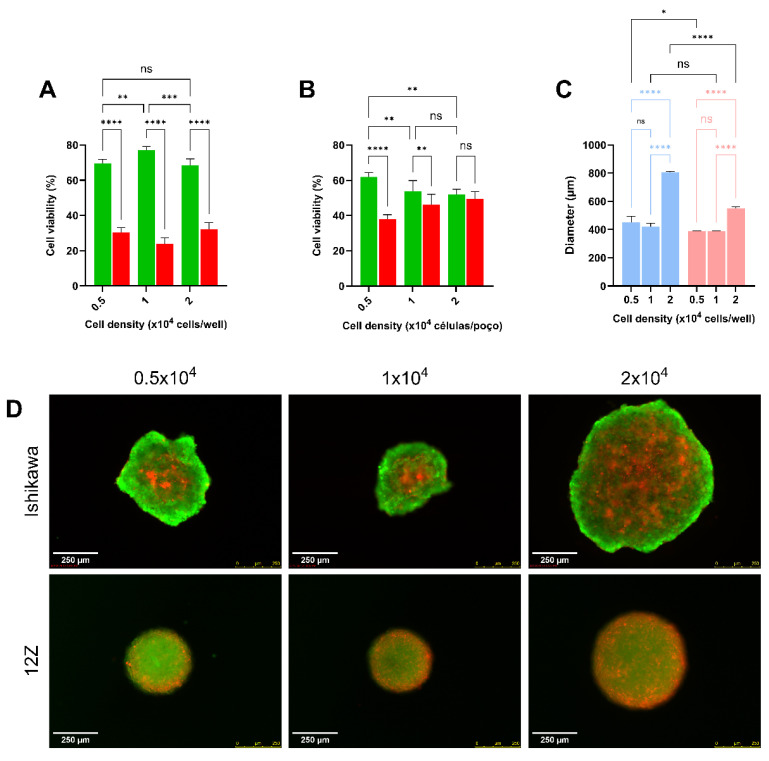
Cell viability and diameter of Ishikawa and 12Z spheroids at different culture densities. Cell viability in 3D culture in Nunclon™ Sphera™ microplates for (**A**) Ishikawa and (**B**) 12Z cells. (**C**) Spheroid diameter. Color scheme: green—live cells; red—dead cells; light blue—Ishikawa 3D culture; salmon—12Z 3D culture. (**D**) Representative images (LIVE/DEAD™: live cells in green; dead cells in red). Scale bar: 250 µm. Data are presented as mean ± SD. Statistical analysis was performed by one-way ANOVA, followed by a Bonferroni post hoc test, comparing all groups. ns = not significant, *, **, ***, and **** Statistically significant difference between groups (*p* < 0.05, 0.01, 0.001, and 0.0001, respectively). Experiments were performed in triplicate as independent assays.

The use of H_2_O_2_ as an oxidative stress inducer for in vitro studies is well described in the literature [38,39]. Before evaluating the effects caused in biomolecules by H_2_O_2_, the cellular density of cells for spheroids construction to achieve recommended size (400 µm) (Figure 1), as well as the toxicity of H_2_O_2_ in bi- and tri-dimensional cell cultures for 12Z and Ishikawa cell lines, were determined by MTT assay and by LIVE/DEAD™ Cell Imaging Kit (Invitrogen), for each cell culture model, respectively. For a 3D tissue-like model that recapitulates key architectural features relevant to endometrial and endometriosis-related in vitro studies, the ideal condition for spheroids establishment is required for both endometrial cell lines [16,40,41] was a 48 h incubation of 0.5 × 10^4^ cells per well in Nunclon™ Sphera™ (Figure 1).

**Figure 2:**
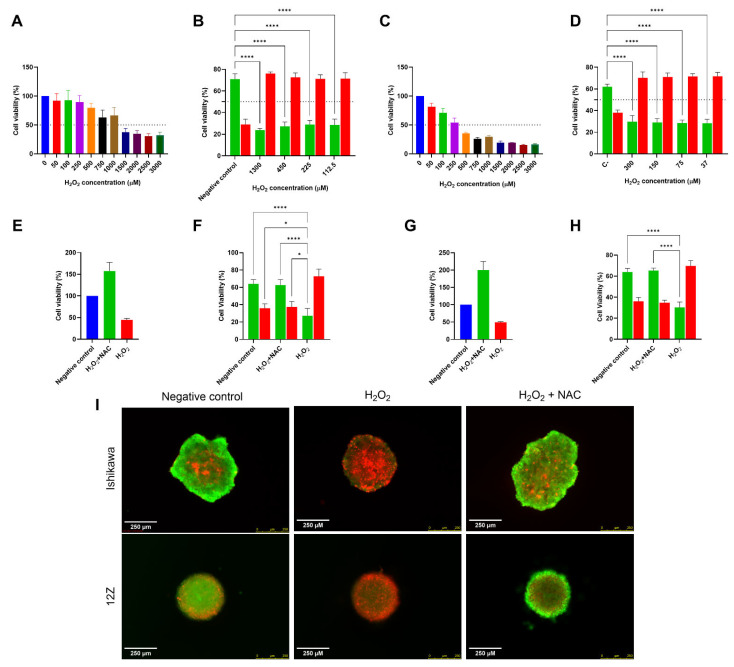
Cytotoxicity of H_2_O_2_ and antioxidant potential of NAC in Ishikawa and 12Z cells in 2D and 3D cultures. Cytotoxicity of H_2_O_2_ in Ishikawa cells in (**A**) 2D and (**B**) 3D cultures and in 12Z cells in (**C**) 2D and (**D**) 3D cultures. Protective effect of NAC in Ishikawa cells in (**E**) 2D and (**F**) 3D cultures and in 12Z cells in (**G**) 2D and (**H**) 3D cultures. Color scheme for (**B**,**D**,**F**,**H**): green—live cells; red—dead cells. (**I**) Representative images (LIVE/DEAD™: live cells in green; dead cells in red). Scale bar: 250 µm. Data are presented as mean ± SD. Statistical analysis was performed by one-way (**A**–**D**,**F**,**H**) and two-way (E, G) ANOVA, followed by a Bonferroni post hoc test, comparing all groups to the control (**B**,**D**) and the H_2_O_2_ treatment (**F**,**H**). * and **** Statistically significant difference between groups (*p* < 0.05 and 0.0001, respectively). Experiments were performed in triplicate as independent assays.

In 2D cultures, with concentrations ranging from 50 to 3000 µM, it was possible to observe that Ishikawa cells exhibited higher tolerance to H_2_O_2_, showing an Effective Concentration 50% (EC_50_) of 1300 ± 148.5 µM, while 12Z cells were more sensitive, presenting an EC_50_ of 296 ± 35.5 µM (Figure 2A,C). These EC_50_ results reveal a differential sensitivity greater than fourfold between the two cell lines, supporting their use for comparative oxidative stress analysis. The cell viability results from 2D cultures guided the selection of the concentrations subsequently assessed in the spheroids. The following H_2_O_2_ concentrations were assessed for Ishikawa and 12Z spheroids, respectively: 112.5, 225, 450 and 1300 µM, and 37, 75, 150 and 300 µM. Similar results for H_2_O_2_ EC_50_ were observed across all concentrations (LIVE/DEAD™ of approximately 30/70%), so we used the lowest peroxide concentration (112.5 µM for Ishikawa and 37 µM for 12Z) (Figure 2B,D).

### 2.2. NAC Prevents H_2_O_2_-Induced Loss of Viability Without Exhibiting Intrinsic Cytotoxicity

In our hypothesis regarding H_2_O_2_-induced oxidative stress, we selected NAC as a model antioxidant. NAC showed no cytotoxic effects at any of the tested concentrations in either cell line, and when its ability to protect cells from H_2_O_2_ toxicity was evaluated, treatment with 5 mM in 2D cultures and 1.25 mM in 3D cultures, followed by H_2_O_2_ exposure, resulted in cell viability comparable to the negative control, suggesting a strong protective effect of NAC (Figure 2E,G).

The following NAC concentrations were assessed for Ishikawa and 12Z spheroids: 1.25, 2.5, and 5 mM. Equivalent results for NAC’s Minimum Effective Concentration (MEC) were observed across all concentrations (LIVE/DEAD™ of approximately 60/40%), so the lowest effective concentration (1.25 mM) was selected (Figure 2F,H).

### 2.3. 12Z Cells Exhibit Elevated Basal ROS Levels and an Exacerbated Oxidative Response to Exogenous Stress

Once the treatment concentrations were determined, experiments were conducted to verify the generation of ROS and the redox status of the cells. ROS production in 3D cultures was assessed by a sensitive cell-permeable redox probe, 2′,7′-Dichlorodihydrofluorescein diacetate (DCFH-DA), with the positive control 2,3-Dimethoxy-1,4-naphthoquinone (DMNQ), and the evaluation of the Redox Status in 2D and 3D cultures was performed through GSH/GSSG ratio and quantification of total glutathione (×10^−7^ mol/mg protein), indirectly reflecting the activity of the glutathione antioxidant enzyme system. Results from the DCFH-DA assay are expressed as mean gray value, an average gray value within the Region of Interest (ROI) of the spheroids, representing the sum of the gray values of all the pixels in the selection divided by the number of pixels.

**Figure 3:**
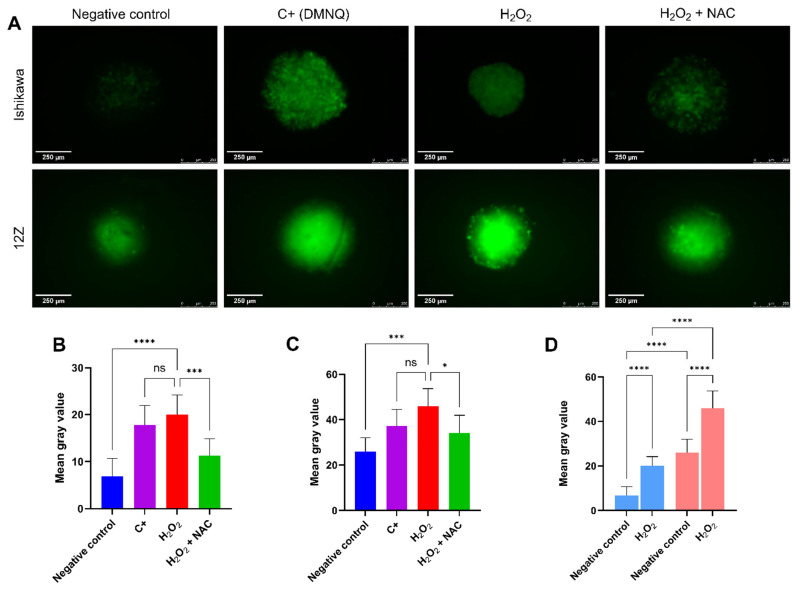
ROS production by DCFH-DA probe in spheroids of Ishikawa and 12Z cell lines. (**A**) Representative images. Mean gray value of (**B**) Ishikawa and (**C**) 12Z spheroids. (**D**) Comparison between groups. Scale bar: 250 μm. Color scheme for D: light blue—Ishikawa 3D culture; salmon—12Z 3D culture. Data are presented as mean ± SD. Statistical analysis was performed by one-way ANOVA, followed by a Bonferroni post hoc test, comparing all groups to the H_2_O_2_ treatment. ns = not significant, *, ***, and **** Statistically significant difference between groups (*p* < 0.05, 0.001, and 0.0001, respectively). Experiments were performed in triplicate as independent assays.

In the negative control, 12Z cells produced approximately four times more ROS than Ishikawa cells (Figure 3D). When observing the different responses between the negative control and the induced oxidative stress, Ishikawa spheroids showed a three-fold increase in mean gray value (Figure 3B), and 12Z spheroids showed an increase of approximately two times (Figure 3C).

Regarding the incubation of the H_2_O_2_ stressor with the antioxidant, NAC showed an inhibitory effect, apparently reducing the presence of ROS in both cell lines, although this reduction occurred more considerably in Ishikawa cells since the statistical difference between the negative control and the treatment with both H_2_O_2_ and NAC was more significant than for the same condition in 12Z (Figure 3B,C).

### 2.4. Glutathione Redox Homeostasis Is Differentially Disrupted by H_2_O_2_ in 12Z and Ishikawa Cells

**Figure 4:**
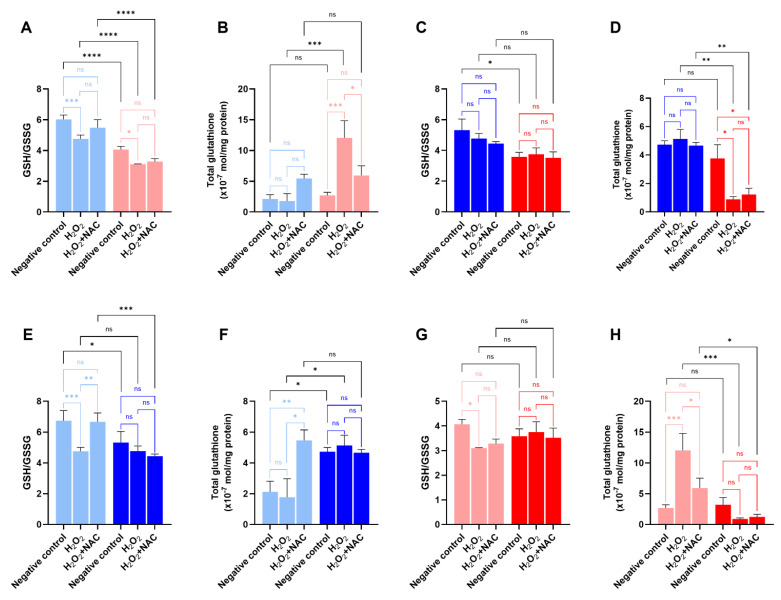
Redox status and total glutathione in 2D and 3D Ishikawa and 12Z cell cultures. (**A**,**C**,**E**,**G**) GSH/GSSG ratio comparing cell lines and culture systems. (**B**,**D**,**F**,**H**) Quantification of total glutathione normalized per mg of protein comparing cell lines and culture systems. Color scheme: light blue—Ishikawa 2D culture; blue—Ishikawa 3D culture; salmon—12Z 2D culture; red—12Z 3D culture. Data are presented as mean ± SD. Statistical analysis was performed by two-way ANOVA, followed by a Bonferroni post hoc test, comparing all groups. ns = not significant, *, **, ***, and **** Statistically significant difference between groups (*p* < 0.05, 0.01, 0.001, and 0.0001, respectively). Experiments were performed in triplicate as independent assays.

The activity of the glutathione antioxidant system was assessed by measuring the classic redox index GSH/GSSG [42,43] and total glutathione levels, quantified and expressed as a normalized value (×10^−7^ mol/mg protein) [44,45]. When comparing the different cell lines in 2D culture, even though the GSH/GSSG ratio was lower for 12Z cells regardless of the treatment, demonstrating a naturally more fragile redox status, 12Z cells showed the same overall trend as Ishikawa cells: lower activity of the antioxidant system in the negative control when compared to the H_2_O_2_ + NAC treatment (negative control: 6.02 in Ishikawa vs. 4.06 in 12Z; H_2_O_2_ + NAC: 5.48 in Ishikawa vs. 3.28 in 12Z), and higher activity when treated only with H_2_O_2_ (4.75 in Ishikawa vs. 3.1 in 12Z) (Figure 4A). Total glutathione values reinforce the negative control and H_2_O_2_ + NAC treatment results (negative control: 2.12 × 10^−7^ mol/mg protein in Ishikawa vs. 2.69 × 10^−7^ mol/mg protein in 12Z; H_2_O_2_ + NAC: 5.46 × 10^−7^ mol/mg protein in Ishikawa vs. 5.92 ×10^−7^ mol/mg protein in 12Z), but showed a more notable difference under H_2_O_2_ treatment. When exposed to oxidative stress, 12Z cells exhibited a drastic increase in total glutathione compared to Ishikawa cells (1.77 × 10^−7^ mol/mg protein in Ishikawa vs. 12.05 × 10^−7^ mol/mg protein in 12Z) (Figure 4B).

The GSH/GSSG ratio of the 3D culture showed similarity when comparing the different treatments, suggesting that the redox balance was maintained at negative control levels (Figure 4C,E,G). The total glutathione measurement in the negative control was slightly lower in 12Z spheroids; however, when treated with H_2_O_2_, Ishikawa maintained the amount of total glutathione at negative control levels (similar to 2D culture), as also observed when exposed to H_2_O_2_ + NAC (Figure 4F). For 12Z, the opposite occurs to what happens in its 2D culture: a sharp decrease in total glutathione levels with both H_2_O_2_ and H_2_O_2_ + NAC (Figure 4H).

### 2.5. Oxidative Stress Preferentially Drives Lipid Peroxidation and Protein Carbonylation in 12Z Cells

**Figure 5:**
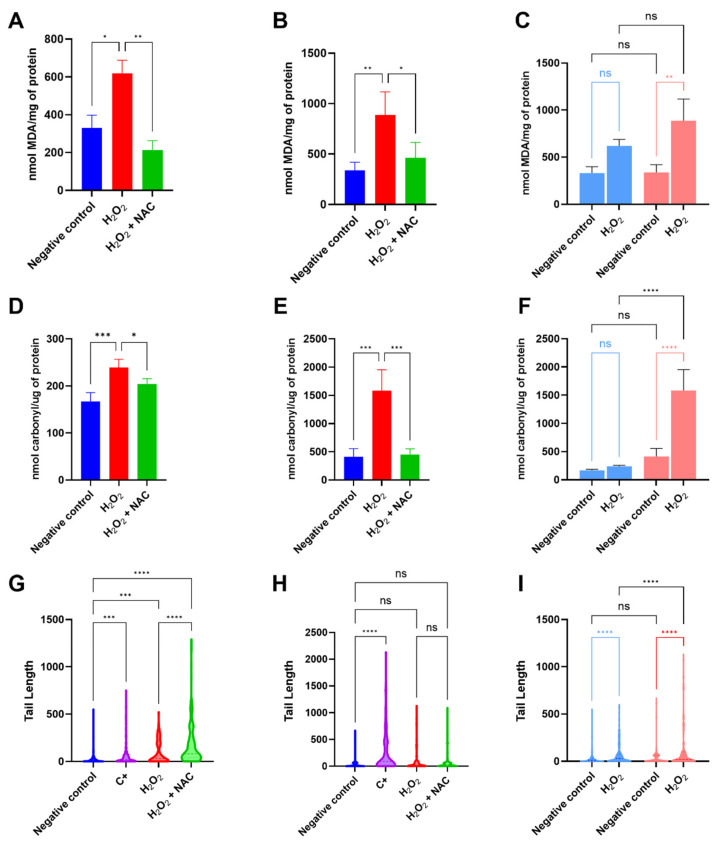
Evaluation of lipid, protein, and DNA damage in 2D culture of Ishikawa and 12Z cells. Lipid peroxidation in (**A**) Ishikawa and (**B**) 12Z 2D cultures, (**C**) and comparison between cell lines. Protein oxidation in (**D**) Ishikawa and (**E**) 12Z 2D cultures, (**F**) and comparison between cell lines. DNA damage analysis via Comet Assay in (**G**) Ishikawa and (**H**) 12Z 2D cultures, (**I**) and comparison between cell lines. Color scheme for (**C**,**F**,**I**): light blue—Ishikawa 2D culture; salmon—12Z 2D culture. Data are presented as mean ± SD, and statistical analysis was performed by two-way ANOVA, followed by Bonferroni post hoc test, comparing all groups to H_2_O_2_ (**A**,**B**,**D**,**E**), to the control (**G**,**H**), and among themselves (**C**,**F**,**I**). ns = not significant, *, **, ***, and **** Statistically significant difference between groups (*p* < 0.05, 0.01, 0.001, and 0.0001, respectively). Experiments were performed in triplicate as independent assays.

Levels of lipid peroxidation in 2D cultures were assessed through the Thiobarbituric Acid Reactive Substances (TBARS) assay. Our results indicate no significant difference in the negative control when comparing cell lines. However, when cells were exposed to H_2_O_2_, the concentration of MDA produced in 12Z cells increased threefold (974.9 nmol of MDA/mg of protein), indicating that it is more sensitive to induced oxidative stress when compared to Ishikawa cells (619.5 nmol of MDA/mg of protein). Our results also corroborate the protective effect of NAC against H_2_O_2_-induced damage, since cells incubated previously with NAC presented MDA concentrations similar to those of untreated cells for both cell lines (Figure 5A–C).

The levels of carbonylated proteins in 2D cultures were determined by the reaction of carbonyl groups with 2,4-dinitrophenylhydrazine (DNPH). Our results indicate that in basal conditions, 12Z cells present a higher concentration of carbonylated proteins in comparison to Ishikawa cells. When exposed to oxidative stress induced by H_2_O_2_, Ishikawa cells did not present changes in levels of carbonylated proteins, while 12Z cells showed a four-fold increase in carbonyl content. It was also possible to observe that NAC inhibits the production of carbonylated proteins in cells previously protected (Figure 5E,F).

### 2.6. 12Z Cells Undergo Severe DNA Damage Following H_2_O_2_ Exposure That Is Not Reversed by NAC

**Figure 6:**
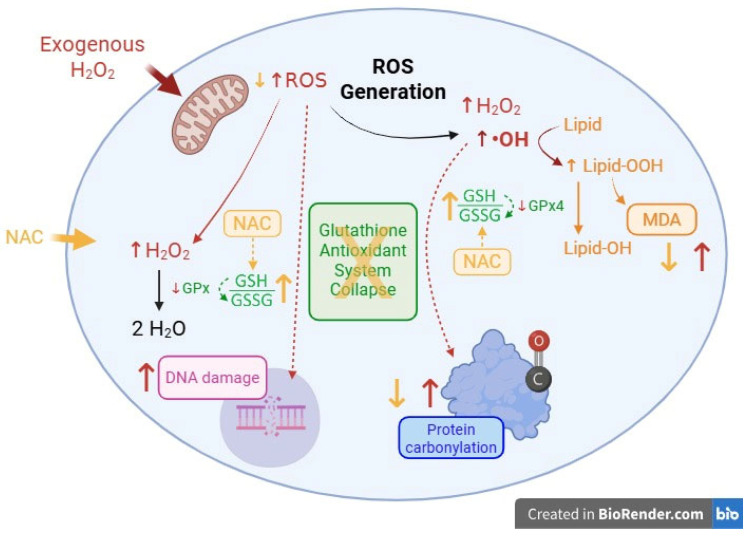
Schematic Representation of Proposed Redox Imbalance and Glutathione Antioxidant System Collapse in 12Z cells under oxidative stress conditions induced by H_2_O_2_. Exogenous H_2_O_2_ increases intracellular ROS levels, potentially leading to the generation of highly reactive hydroxyl radicals (•OH). The impaired glutathione peroxidase (GPx and GPx4) activity leads to glutathione antioxidant system failure (GSH/GSSG imbalance), resulting in lipid peroxidation (lipid-OOH), MDA accumulation, protein carbonylation and DNA damage. NAC partially mitigates ROS production and restores cell viability but does not prevent oxidative DNA damage. Created with BioRender.

The Comet Assay was used to assess DNA damage in 2D cultures. Results indicated that Ishikawa cells exhibited higher resistance to oxidative challenge, as observed for the positive control (DMSO 20%*_v_*_/*v*_) and H_2_O_2_ treatment, in comparison to 12Z cells, presenting a Tail Length (pixels) of 750 and 600, for each treatment, respectively, while 12Z cells showed values of 2250 and 1150 under the same conditions. Despite NAC being able to preserve cell viability under H_2_O_2_ exposure, it did not reduce DNA damage (Figure 5G–I). All the results presented here are summarized in Figure 6, and the numerical values are available in Supplementary Table S1, Figure S1.

## 3. Discussion

Since 1920, several hypotheses about the origins of endometriosis have been discussed [46,47,48,49], and more recent studies have identified inflammation as a hallmark of the disease, particularly mechanisms linked to oxidative stress [50,51,52,53]. Despite remaining incompletely characterized, the imbalance between oxidants and antioxidants is strongly implicated in endometriosis-associated pain [54,55,56]. Based on this framework, the present study evaluated and compared the effects of H_2_O_2_-induced oxidative stress on the biochemical profile of two widely used in gynecological epithelial cell lines in vitro: Ishikawa, an endometrial adenocarcinoma cell line that retains several characteristics of endometrial epithelium and was therefore used here as a comparator; and 12Z cell line, an *N*-cadherin-positive human endometrial cell line widely used in endometriosis-related in vitro studies, including investigations of invasion, cellular behavior, and therapeutic responses [40,57,58].

Consistent with our hypothesis, 12Z cells proved to be more sensitive to H_2_O_2_. The EC_50_ values obtained for Ishikawa cells are comparable to previously reported data [37]. NAC, a widely employed antioxidant, acts through several mechanisms, including GSH replenishment, H_2_O_2_ scavenging, and Nrf2 pathway modulation [28,29]. Consistent with its known properties, NAC served as an effective positive control in our models, restoring cell viability after H_2_O_2_ exposure to levels comparable to the negative control, and increasing viability in both cell lines. The LIVE/DEAD™ assay for 3D cultures corroborated MTT results. However, the limited discrimination between H_2_O_2_ concentrations suggests reduced assay sensitivity, due to limited dye penetration into the spheroid core and the heterogeneous diffusion of reagents [59,60]. Thus, it did not demonstrate a dose-dependent response to H_2_O_2_ treatment, but the assay remained adequate for defining experimental conditions.

Baseline fluorescence observed in negative controls is consistent with the intrinsic oxidation dynamics of the DCFH-DA probe, which is further enhanced under oxidative conditions [61]. The variation in 12Z when exposed to H_2_O_2_ is low compared to its high basal level of oxidative stress [2,18,62], may reflect adaptive mechanisms that limit the additional impact of exogenous oxidative challenge. In contrast, Ishikawa cells displayed lower ROS levels across all conditions, highlighting intrinsic differences in redox status between the two cell lines. When treated simultaneously with H_2_O_2_ and NAC, the antioxidant showed an inhibitory effect on H_2_O_2_-induced oxidative stress. These results are consistent with reports of enhanced ROS generation in biological samples from patients with endometriosis [14,15,17,63].

Regarding 2D cultures, 12Z cells exhibited a lower GSH/GSSG ratio than the Ishikawa cells, though both followed the same tendencies, with NAC restoring redox balance and protecting the GSH pool [28,29]. Notably, H_2_O_2_ exposure triggered a pronounced increase in total glutathione exclusively in 12Z cells, suggesting an exacerbated compensatory response [64]. This likely reflects the immortalized nature of the model [62,65,66], potentially corresponding to an early adaptive response rather than a sustained physiological state [62]. The 3D microenvironment provides a more constrained and physiologically relevant context for redox regulation, as cells prioritize the GSH/GSSG ratio for survival over the total glutathione pool. Although negative control spheroids maintained higher total glutathione than 2D cultures due to increased antioxidant demand [67], their response to H_2_O_2_ differed significantly: while Ishikawa spheroids preserved their levels, 12Z spheroids showed a prominent decrease. This indicates a collapse of the antioxidant system in 12Z spheroids under oxidative stress conditions; while GR activity sustains the GSH/GSSG ratio via recycling, it incurs a high metabolic cost, evidenced by a 72% depletion of total glutathione [64,68,69]. This underscores the robust but energetically costly antioxidant response of 12Z cells, achieved through GSH pool exhaustion or compensatory mechanisms like CAT and SOD [2,21].

Lipid peroxidation is expected to be higher in cellular systems associated with endometriosis-related oxidative stress [70,71,72,73,74,75], a process largely mitigated by GPx4 through the reduction in lipid hydroperoxides to nontoxic alcohols using the glutathione system [62,64,76,77,78,79,80,81]. TBARS assays revealed comparable and lower lipid damage in the negative control and H_2_O_2_ + NAC groups for both cell lines, consistent with a high GSH/GSSG ratio, lower GPx4 demand, and lower total GSH. However, isolated H_2_O_2_ exposure tripled MDA levels in 12Z cells and doubled them in Ishikawa cells compared to controls, confirming the greater sensitivity of 12Z to induced oxidative stress. This aligns with observed GSH oxidation and the drastic increase in total glutathione in 12Z cells, indicating compensatory redox hyperactivation. Consistent with lipid peroxidation patterns, H_2_O_2_ exposure induced a moderate but significant increase in PCO levels in Ishikawa cells, only partially reversed by NAC—potentially due to limited uptake or alternative oxidative pathways [29,37]. In contrast, 12Z cells exhibited a significantly more pronounced PCO increase upon H_2_O_2_ treatment. Notably, NAC co-treatment effectively restored PCO levels to near-control levels. This suggests that 12Z cells possess a higher susceptibility to oxidative damage but also a greater reliance on redox-sensitive mechanisms that are highly responsive to NAC supplementation [50,82].

DNA damage analysis revealed minimal DNA damage in untreated controls of both cell lines. However, upon exposure to H_2_O_2_, 12Z cells exhibited greater DNA fragmentation compared to Ishikawa cells, indicating heightened susceptibility to oxidative stress-induced damage. Despite NAC’s efficacy in other biochemical assays, it failed to attenuate H_2_O_2_-induced DNA damage. This outcome may reflect the contribution of iron-dependent oxidative processes described in endometriosis, which are not directly modeled in the present system, as well as NAC’s limited capacity for iron chelation and •OH neutralization [83]. Recent studies highlight that iron-driven oxidative reactions contribute to genomic instability and ferroptosis processes in endometriosis [84,85]. These findings highlight the limitations of conventional antioxidant strategies in iron-enriched oxidative environments [86,87,88].

A major limitation of this study lies in the use of immortalized cell lines, which lack the multicellular and inflammatory complexity of endometriosis lesions in vivo. Additionally, the adenocarcinoma origin of Ishikawa cells may contribute to increased oxidative stress resistance due to cancer-associated redox adaptations, which should be considered when interpreting the differences observed between cell lines. Therefore, future studies employing primary cultures, organoids, or ex vivo tissues will be essential for translational validation. This study was designed as a controlled in vitro investigation of oxidative stress mechanisms and did not aim to directly model the full pathophysiology of endometriosis or to provide clinical or therapeutic conclusions. Despite these limitations, from a mechanistic perspective, these findings reinforce oxidative stress as a vital component of endometriosis pathophysiology. 12Z cells displayed intrinsic redox fragility, exaggerated biomolecular damage, and incomplete antioxidant rescue under oxidative challenge. Consistent with this, the 3D spheroid model revealed a pronounced depletion of total glutathione in 12Z cells under oxidative stress, indicative of redox exhaustion and supporting its application in controlled mechanistic studies.

## 4. Materials and Methods

### 4.1. General Procedures

All reagents were obtained from Sigma-Aldrich and used without further purification. NAC and H_2_O_2_ solutions were prepared with ultrapure MilliQ water (MIliQ, Burlington, MA, USA), and peroxide concentration was determined before each experiment by UV-Vis (UV-1800; Shimadzu Corp., Kyoto, Japan) spectroscopy as reported in the literature [89].

### 4.2. Cell Culture

Immortalized Ishikawa endometrial epithelial adenocarcinoma cells (ECACC 99040201) and human endometriotic epithelial 12Z cells (SCC443) were purchased from Sigma-Aldrich (St Louis, MO, USA) and maintained at 37 °C in a humidified atmosphere with 5% CO_2_. Ishikawa cells were cultured in MEM supplemented with 5% FBS, while 12Z cells were cultured in High-Glucose DMEM supplemented with 10% FBS. Both media were supplemented with penicillin (100 U/mL), streptomycin (10 μg/mL), non-essential amino acids, and sodium pyruvate.

For 3D spheroid formation, cells were seeded in 96-well Nunclon^TM^ Sphera^TM^ U-bottom low-adhesion plates (Thermo Scientific, Waltham, MA, USA) and incubated for 48 h. Based on literature guidance [41], seeding densities of 0.5, 1.0, and 2.0 × 10^4^ cells/well were evaluated to obtain spheroids with minimal baseline cell death (negative control) and diameters of 400–600 μm [41]. Spheroid morphology was assessed by inverted microscopy prior to the experiments.

### 4.3. Cell Viability Assay

Cell viability in 2D cultures was assessed by the MTT assay [90]. Ishikawa and 12Z cells were seeded in 96-well plates (4 × 10^4^ cells/cm^2^) and allowed to attach for 24 h. Cells were then exposed to H_2_O_2_ (50–3000 μM) for 24 h. After treatment, MTT solution (5 mg/mL; 30 μL/well) was added, and plates were incubated for 2 h at 37 °C and 5% CO_2_, protected from light. Cell viability was calculated relative to untreated controls, and EC_50_ values were obtained by non-linear regression in GraphPad Prism 10.0. All experiments were performed in triplicate as independent assays.

NAC cytotoxicity was evaluated using the same protocol by treating cells with NAC (1, 5 and 10 mM) for 24 h, with results expressed as percentage viability relative to untreated controls. Based on these data, the protective effect of NAC against H_2_O_2_ was determined by pre-treating cells with NAC (5 mM) for 10 min, followed by incubation with H_2_O_2_ at the previously defined concentrations for 24 h. Untreated cells were used as negative controls and H_2_O_2_-treated cells as positive controls. Viability was measured by MTT as previously described.

The concentration of H_2_O_2_ and NAC selected in 2D was subsequently used to guide treatments in spheroids. Viability in 3D cultures was measured using the LIVE/DEADTM Cell Imaging Kit (Calcein-AM/BOBO-3 iodide; Invitrogen^TM^, Carlsbad, CA, USA) diluted 1:1 in HBSS (pH 7.5) and added at 50 μL/well. Fluorescence images were acquired on a Leica AF6000 microscope (Wetzlar, Germany) (λ_Ex/Em_ 488/515 nm for Calcein-AM; 570/602 nm for BOBO-3 iodide). Images were analyzed in ImageJ V1.54 by calculating corrected total cell fluorescence (CTCF = integrated density − [area × mean background]). For each fluorophore, CTCF values from all fields were summed to obtain total fluorescence (CTCF_total), and viability was expressed as [CTCF_individual/CTCF_total] × 100. EC_50_ values were determined by non-linear regression in GraphPad Prism 10.0. All experiments were performed in triplicate as independent assays.

### 4.4. Determination of ROS Production by DCFH-DA Staining

ROS production was evaluated by DCFH-DA staining followed by fluorescence microscopy [91,92,93]. DMNQ (100 μM) was used as a positive control for ROS generation, and NAC as an antioxidant control. H_2_O_2_ (EC_50_) and NAC (MEC) concentrations were defined beforehand using the LIVE/DEAD^TM^ assay. Untreated spheroids with and without staining served as negative controls.

Spheroids were generated as previously described and then exposed to treatments for 3 h. Sequentially, spheroids were washed with PBS (2×) and incubated with DCFH-DA (50 μM; 200 μL/well) for 45 min at 37 °C protected from light. After two additional PBS washes, fluorescence was recorded on a Leica AF6000 microscope (λ_Ex/Em_ 498/522 nm). Images were quantified in ImageJ, and results are reported as mean gray value. All experiments were performed in triplicate as independent assays.

### 4.5. Determination of the Redox Status by GSH/GSSG Ratio

2D cells were seeded (4 × 10^4^ cells/cm^2^, 25 cm^2^ flasks, 24 h) and spheroids were generated as described above (48 h), followed by 24 h treatments with H_2_O_2_, NAC or H_2_O_2_ + NAC; untreated samples were used as controls.

2D cells were collected by trypsinization, whereas spheroids were lysed by sonication (40% amplitude; cycle 1; 10 s). Lysates were centrifuged (1300 rpm, 5 min, 4 °C) and washed with ice-cold PBS (1500× *g*, 3 min, 4 °C, 3×). Pellets were resuspended in 240 μL of cold ultrapure water and subjected to a freeze–thaw step in liquid nitrogen (−196 °C, 1 min). After thawing, 40 μL were stored at −80 °C for protein quantification (Lowry method). The remaining 200 μL were deproteinized with 50 μL of 10%_w/v_ sulfosalicylic acid and centrifuged (4000× *g*, 5 min, 4 °C); GSH and GSSG were then quantified in the supernatant.

GSH was measured by monitoring the increase in absorbance at 412 nm (DTBN/GR system), and GSSG by monitoring the decrease in NADPH at 340 nm. Concentrations were calculated by the Lambert-Beer law, and redox status was expressed as the GSH/GSSG ratio. Total glutathione was reported as ×10^−7^ mol/mg of protein, with protein levels determined by the Lowry method using BSA as a standard [42,43,45,93,94,95,96,97]. Experiments were performed in triplicate as independent assays.

### 4.6. Determination of DNA Damage by Comet Assay

DNA damage after H_2_O_2_ exposure was assessed by alkaline comet assay with SYBR Gold staining [98,99]. Cells were seeded in 24-well plates (1 × 10^5^ cells/well) for 24 h and then treated for 24 h with H_2_O_2_, NAC or H_2_O_2_ + NAC; untreated cells were used as controls. Cells were collected, washed with PBS, embedded in 1%_w/v_ low-melting agarose, and layered onto slides pre-coated with 1.5%_w/v_ agarose. After solidification at 4 °C, slides were lysed for 1 h at 4 °C (2.5 M NaCl, 100 mM EDTA, 10 mM Tris, 10%_v/v_ DMSO, 1%_v/v_ Triton X-100). Slides were then incubated in alkaline electrophoresis buffer (300 mM NaOH, 100 mM EDTA, pH > 13) for 20 min and electrophoresed at 25 V and 300 mA for 15 min. After electrophoresis, slides were neutralized with Tris buffer (pH 7.5), washed twice with dd-H_2_O, dried at 37 °C for 2 h, and fixed in cold ethanol for 5 min. DMSO at 20% (*v*/*v*) was used as an internal reference for robust DNA damage induction, reflecting cytotoxicity-associated genomic instability rather than a classical genotoxic mechanism.

For staining, slides were incubated with 1× SYBR Gold in TE Buffer for 30 min protected from light, dried at 37 °C for 2 h, and imaged by fluorescence microscopy (λ_Ex/Em_ 496/539 nm). Comet parameters were quantified using the OpenComet plugin in ImageJ. Experiments were performed in triplicate as independent assays.

### 4.7. Determination of Lipid Peroxidation

To assess lipid peroxidation, TBARS were measured as described in the literature [100]. Cells were seeded in 25 cm^2^ flasks (4 × 10^4^ cells/cm^2^) for 24 h, treated as described above, and incubated for an additional 24 h at 37 °C and 5% CO_2_. Cells were washed twice with saline, resuspended in 1 mL of 50 mM sodium phosphate buffer, and stored at −80 °C until analysis. Samples were scraped and transferred to 15 mL tubes; an aliquot (50 μL) was reserved for protein quantification (Lowry method), and the remaining suspension was mixed with 1 mL TBA-TCA reagent. After heating (45 min, boiling water bath) and cooling to room temperature, samples were centrifuged (300× *g*, 5 min) and absorbance was read at 535 nm. MDA equivalents were calculated using ε = 1.49 × 10^5^ L/mol.cm and expressed as nmol MDA/mg of protein. Experiments were performed in triplicate as independent assays.

### 4.8. Determination of Protein Carbonylation

Protein carbonylation was quantified by DNPH derivatization as previously described [100]. Cells were seeded in 25 cm^2^ flasks (4 × 10^4^ cells/cm^2^) for 24 h, treated as previously described, then collected by trypsinization, washed three times with PBS, and lysed in RIPA buffer (100 μL). Lysates were kept on ice for 30 min and centrifuged (14,000 rpm, 20 min, 4 °C). An aliquot (10 μL) was reserved for protein quantification (Lowry method). Volumes corresponding to 1 mg of protein were reacted with DNPH (10 mM in 2.0 M HCl; 500 μL) for 1 h at 37 °C. Proteins were precipitated with 20%_v/v_ TCA (500 μL), centrifuged (11,000 rpm, 5 min, 4 °C), and the pellet was washed three times with EtOH/EtOAc (1:1, 3 × 100 μL). Pellets were then solubilized in 6 M guanidine in 20 mM phosphate buffer (15 min, 37 °C. Carbonyl content was determined at 370 nm (ε = 22 × 10^3^ L/mol.cm) and expressed as nmol of carbonyl/mg of protein. Experiments were performed in triplicate as independent assays.

### 4.9. Statistical Analysis

All experiments were repeated at least three times as independent replicates, and results from cellular assays are presented as mean ± standard deviation. Statistical analyses were performed using ANOVA followed by Bonferroni’s multiple comparisons test. For each comparison, mean differences were reported together with their corresponding 95% confidence intervals and multiplicity-adjusted *p*-value.

## 5. Conclusions

Our findings confirm that 12Z cells exhibit reduced antioxidant capacity and increased susceptibility to oxidative damage compared to Ishikawa cells. This is evidenced by lower EC_50_ values, elevated basal ROS, and significant damage to DNA, lipids, and proteins, patterns that are consistent with oxidative features associated with endometriosis. 12Z cells maintained fragile redox homeostasis, preserving GSH/GSSG ratio under stress despite total glutathione depletion in 3D cultures, a pattern consistent with redox exhaustion under sustained oxidative challenge. Furthermore, while NAC effectively mitigated ROS production and restored cell viability, its inability to prevent DNA damage suggests that oxidative genomic injury in this cellular system involves pathways not directly modulated by NAC’s antioxidant effects.

These data indicate that antioxidant approaches relevant to endometriosis-associated oxidative stress may need to go beyond generic ROS scavenging. The inability of NAC to prevent genomic injury, despite maintaining cell viability, underscores the importance of investigating additional mechanisms contributing to oxidative DNA damage in more complex experimental systems. The antioxidant exhaustion observed in 3D models suggests that metabolic interventions aimed at preserving glutathione homeostasis and genomic stability deserve further investigation.

Ultimately, these results highlight oxidative stress as a key component of the mechanisms associated with endometriosis. Our data support the use of 3D models to better capture cellular metabolic stress under constrained conditions, evidencing glutathione dysregulation as a relevant feature of this microenvironment. These findings, together with iron-mediated ROS pathways well-described in endometriosis but not directly modeled here, represent promising directions for future investigation in endometriosis-related contexts.

## Data Availability

The original contributions presented in this study are included in the article/Supplementary Materials. Further inquiries can be directed to the corresponding author(s).

## Supplementary Materials

The following supporting information can be downloaded at: https://www.mdpi.com/article/10.3390/ijms27094131/s1.

## Abbreviations

8-OHdG	8-hydroxy-2’-deoxyguanosine
ANOVA	Analysis of variance
CAT	Catalase
DCFH-DA	2’,7’-dichlorodihydrofluorescein diacetate
dd-H2O	Double-distilled water
DMEM	Dulbecco’s Modified Eagle’s Medium
DMNQ	2,3-dimethoxy-1,4-naphtoquinone
DNPH	2,4-dinitrophenylhydrazine
EC_50_	Effective Concentration 50%
EtOAc	Ethyl Acetate
EtOH	Ethanol
FBS	Fetal Bovine Serum
GPx	Glutathione peroxidase
GR	Glutathione reductase
GSH	Reduced Glutathione
GSSG	Oxidized Glutathione
HBSS	Hank’s Balanced Salt Solution
MDA	Malondialdehyde
MEC	Minimum Effective Concentration
MEM	Minimum Essential Medium
MTT	3-(4,5-dimethylthiazol-2-yl)-2,5-diphenyltetrazolium bromide
NAC	*N*-acetylcysteine
NF-κB	Nuclear Factor Kappa-light-chain-enhancer of activated B cells
Nrf2	Nuclear Factor E2-related factor 2
PBS	Phosphate Buffered Saline
PCO	Protein Carbonyls
PUFAs	Polyunsaturated Fatty Acids
RIPA	Radioimmunoprecipitation Assay Buffer
ROI	Region of Interest
ROS	Reactive Oxygen Species
SOD	Superoxide Dismutase
TBA	Thiobarbituric Acid
TCA	Trichloroacetic Acid
TBARS	Thiobarbituric Acid Reactive Substances
TE	Tris-EDTA Buffer
