# Supplementary material for: Differential Antioxidant Capacities of Human Endometriotic and Endometrial Cell Models Under H2O2 Exposure
Source: Int J Mol Sci. 2026 May 5;27(9):4131. doi: 10.3390/ijms27094131 (PMC13164405; doi:10.3390/ijms27094131)
Supplement: Supplementary file 1 [file ijms-27-04131-s001.zip › ijms-4196167-supplementary.pdf]

Supplementary Information

Differential Antioxidant Capacities of Human Endometriotic and Endometrial Cell Models Under H_2O_2 Exposure

Julia A. Coelho, Kaio S. Gomes * and Giselle Cerchiaro *

Metal Biochemistry and Oxidative Stress Laboratory, Centre for Natural Sciences and Humanities, Federal University of ABC, Santo Andre 09280-560, SP, Brazil
*Correspondence: kaio.souza@ufabc.edu.br (K.S.G.); giselle.cerchiaro@ufabc.edu.br (G.C.); Tel.: +55-1149960043 (K.S.G. & G.C.)

1. Experimental Session

a. General Procedures

All reagents were obtained from Sigma-Aldrich and used without further purification. NAC and H_2O_2 solutions were prepared with MiliQ water, and peroxide concentration was determined before each experiment by UV-Vis spectroscopy as reported in the literature [1].

b. Cell Culture

Immortalized Ishikawa endometrial epithelial adenocarcinoma (ECACC 99040201) and human endometriotic epithelial 12Z (SCC443) cell cultures were obtained from Sigma-Aldrich, and cultured at 37 ºC and 5% CO_2 in 75 cm^2 culture flask in MEM medium supplemented with 5% of FBS and DMEM High Glucose medium with 10% FBS, respectively, with addition of antibiotic solution (100 U/mL of penicillin and 10 U/mL of streptomycin), non-essential amino acids and sodium pyruvate .

To establish 3D cultures, the tested cell densities were determined as reported in the literature [2], i.e., $2x10^4$ cells per well for 48 hours when generated in low-adhesion microplates. Thus, the cells were incubated for 48 hours in a 96-well Nunclon™ Sphera™ U-shaped bottom microplate (ThermoScientific).

Cell densities of 0.5, 1 and $2x10^4$ cells/well were tested, seeking the one that resulted in spheroids with a low amount of death in the negative control (only culture medium) and with a diameter between 400-600 µm, which, according to the literature, is the ideal size to mimic endometriotic lesions [2]. The morphology of the spheroids was analyzed by inverted microscope prior to the tests.

c. Cell Viability Assay

Cell viability was determined by MTT assay [3]. Ishikawa and 12Z cells were placed on 96-well plates at density of $4x10^4$ cell/cm^2 for 24 h, sequentially cells were treated with solution of H_2O_2 ranging from 3000 to 50 µM were added, incubated for 24h and afterward, 30 mL of MTT solution (5 mg/mL) was added to each well, and plates kept covered from light at 37 ºC, 5% CO_2 for 2 h. EC_{50} values were determined by non-linear regression using the software GraphPad Prism 10.0. All experiments were realized in triplicate as independent assays.

To evaluate the toxicity of NAC, the same procedure was applied, treating the cells with NAC at 10, 5 and 1 mM for 24 h and results were expressed as Cell Viability (%) in comparison to the negative control (untreated cells).

After assessment of NAC cytotoxicity, its protective effect against H_2O_2 was also determined by MTT assay. Cells were treated with 5 mM of NAC 10 min, then incubated with H_2O_2 at previously determined concentrations. After 24 h of incubation, the cell viability was measured as previously described and results were expressed as Cell Viability (%) in comparison to the negative control (untreated cells). Cells treated with H_2O_2 were used as positive control.

The results of cell viability in 2D cultures were used comparatively to define the concentrations of H_2O_2 and NAC evaluated for the spheroids. The viability analysis of the 3D cultures was performed using 50µL/well of the LIVE/DEAD™ Cell Imaging Kit (488/570, Calcein-AM/BOBO-3 iodide, Invitrogen™) in 1:1 HBSS (pH 7.5). Fluorescence levels were measured using Leica AF6000 fluorescence microscope ($l_{EX/EM}$ 488/515 nm for Calcein-AM, $l_{EX/EM}$ 570/602 nm for BOBO-3 iodide). Images were analyzed using ImageJ software to determine the Corrected Total Cell Fluorescence (CTCF = Integrated Density – [Area × Mean background fluorescence]). For each fluorophore, the CTCF obtained from all filters was summed to calculate the total fluorescence (CTCF_total), and the viability percentage was expressed as the ratio of individual CTCF to the total fluorescence ([CTCF_individual / CTCF_total] × 100). EC_{50} values were

determined by non-linear regression using the GraphPad Prism 10.0 software. All experiments were realized in triplicate as independent assays.

d. Determination of ROS Production by DCFH-DA Staining

The presence of ROS after induction of oxidative stress by H_2O_2 was evaluated by the reaction of ROS with the sensitive cell-permeable redox probe, DCFH-DA, assessed by fluorescence microscopy [4–6]. The positive control for ROS generation was DMNQ 100μM and NAC as antioxidant. The EC_{50} H_2O_2 and IC_{50} NAC concentrations were predetermined using LIVE/DEAD™. The negative controls were untreated cells with and without staining.

For 3D culture, the spheroids were cultured for 48 hours in 96-well Nunclon™ Sphera™ at an adequate cellular density, as predetermined by LIVE/DEAD™ assay. The spheroids were incubated with the treatments as well as DMNQ and negative controls for 3 h. Samples were washed twice with PBS and incubated with 200 μL of 50 μM DCFH-DA solution at 37 ºC for 45 min kept in the absence of light. Then, samples were washed twice with PBS and fluorescence levels were measured using Leica AF6000 fluorescence microscope ($l_{EX/EM}$ 498/522 nm). The images obtained were analyzed using ImageJ software. Experiments were conducted in triplicate as independent assays, and results are expressed as mean grey value.

e. Determination of the Redox Status by GSH/GSSG ratio

For 2D culture, endometrial cells were plated $4x10^4$ cells/cm^2 in 25 cm^2 culture flasks for 24 h. For 3D culture, spheroids were plated in 96-well Nunclon™ Sphera™ at the appropriate cell density for 48 h, as predetermined by the LIVE/DEAD™ assay. The cultures were then incubated for 24 h with the treatments, i.e., negative control (untreated cells), oxidant (H_2O_2), antioxidant (NAC), and the combination of both.

For cell lysis, cells in 2D cultures were trypsinized, and spheroids were lysed through sonication (amplitude 40%, cycle 1, 10 s). Next, cells were centrifuged (1,300 rpm for 5 minutes at 4ºC) and washed three times with ice-cold PBS (1,500xg for 3 minutes at 4 ºC). The cells were then resuspended in 240 μL of cold ultra-pure water and lysed by rapid freezing in liquid nitrogen (-196 ºC, 1 min). After thawing, 40 μL of the cell suspension were stored at -80ºC for subsequent protein quantification using Lowry method. Finally, 50 μL of 10% $_{w/v}$ sulfosalicylic acid solution was added to the remaining 200 μL of cell suspension and another centrifugation was performed (4,000xg for 5 minutes at 4 ºC), and the levels of GSH and GSSG in the supernatant were assessed using a previous protocol [7–9].

For GSH quantification, the assay mixture contained 100 μL of supernatant, 790 μL of a 0.1 M sodium phosphate buffer containing 0.05% EDTA at pH 7.0, 100 μL of 6 mM of DTNB dissolved in glutathione assay buffer (GAB; 125 mM sodium phosphate containing 6.3 mM EDTA) and 10 μL glutathione reductase (55 U/ml). For GSSG quantification, the assay mixture comprised 100 μL of supernatant, 190 μL of 0.5 M phosphate buffer at pH 6.8, 700 μL of 0.3 mM NADPH prepared in GAB, and 10 μL of glutathione reductase (55 U/ml). The reaction rate was estimated from the change in absorbance at 412 nm after 3 min at 25ºC (for GSH) or at 340 nm after 16 min at 30ºC (for GSSG).

The accuracy of the GSH reference standard was measured with DTNB using a molar extinction coefficient of 13600 L/mol.cm with an absorbance of 412 nm [10]. GSSG was standardized by measuring the decrease in NADPH in the presence of glutathione reductase, taking into consideration a molar extinction coefficient of NADPH of 6,270 L/mol.cm at 340 nm, with 1 mol of NADPH converting 1 equivalent of GSSG to 2 equivalents of GSH [11].

The ratio of concentrations (nmol/L) of reduced/oxidized glutathione (GSH/GSSG), proposed as a sensitive redox index [12,13], was determined by spectrophotometric quantification with concentrations derived from the Lambert–Beer law. Total glutathione levels were expressed as x10^{-7} mol per mg of protein, with protein content determined by the Lowry method using BSA as standard.

f. Determination of DNA Damage by Comet Assay

To evaluated DNA damage caused by exposure to H_2O_2, Comet Assay with Sybr Gold staining was performed [14,15]. Cells were placed on 24-well plates at density of 1x10^5 cells/well and incubated for 24 h at 37 ºC and 5% CO_2, then treated with H_2O_2, NAC and both solutions at concentrations previously determined for 24 h. Then, cells were detached, washed with PBS, resuspended with 100 mL of agarose low-melting 1%$_{w/v}$, placed in microscope slides pre-covered with agarose 1.5% $_{w/v}$, covered with microslides, and allowed to solidify at 4 ºC. Afterward, the microslides were removed and slides incubated in cold lysis solution (2.5 M NaCl, 100 mM EDTA, 10 mM TRIS, 10%$_{v/v}$ DMSO, 1%$_{v/v}$ Triton X-100) for 1 h. After this time, the slid/es were placed on the horizontal electrophoresis tank with electrophoretic solution (300 mM NaOH, 100 mM EDTA, pH>13) and incubated for 20 min. The horizontal electrophoresis was performed with 25 V and 300 mA for 15 min, then the slides were neutralized with Tris buffer (pH 7.5), washed with dd-H_2O twice, dried at 37 ºC for 2 h, and fixed with cold EtOH for 5 min.

To stain the cells, slides were placed in the staining container and 25 mL of 1x SYBR Gold in TE buffer (0.5 M Tris-HCl, 200 mM EDTA, pH 7.5) were added, the containers were agitated for 30 min covered from light, then the slides were dried at 37 ºC for 2 h covered from light and analyzed by fluorescence microscopy (l$_{EX/EM}$ 496/539 nm). Images were analyzed with the plugin OpenComet for ImageJ.

g. Determination of Lipid Peroxidation

To evaluate the levels of peroxidized lipids, TBARS assay was performed [16]. Cells were plated at a density of 4x10^4 cells/cm^2 in 25 cm^2 culture flasks for 24 h. Then, treated as previously described and, after incubation of 24 h at 37 ºC and 5% CO_2, cells were washed twice with saline solution and frozen in 1 mL Sodium Phosphate Buffer (50 mM) for 24 h. After this time, cells were scraped, and the suspension was transferred to 15 mL conical tubes. 50 mL of each sample was separated for protein quantification by Lowry assay, and to the remaining, 1 mL of TBA-TCA solution was added. The mixture was boiled for 45 min and allowed to warm to room temperature, then centrifuged for 5 min at 300 xg. The absorbance of supernatant was measured at 535 nm and the concentration of TBA-MDA complex was determined (e = 1.49x10^5 L/mol.cm). Results were expressed in nmol of MDA/mg of protein and experiments were conducted in triplicate as independent assays.

h. Determination of Protein Carbonylation

The concentration of carbonylated proteins was measured by the reaction of carbonyl products with DNPH [16]. Cells were placed on 25 cm^2 culture flasks at a density of 4x10^4 cells/cm^2 and incubated for 24 h at 37 ºC, 5% CO_2, then treated as in previous experiments. Then, cells were trypsinized, washed with PBS (3x) and resuspended in 100 mL of RIPA. Samples were kept in an ice bath for 30 min, and centrifuged at 4 °C, 14,000 RPM, for 20 min. 10 mL of each sample was separated for protein quantification by the Lowry method. Afterwards, to volumes equivalent to 1 mg of protein were added 500 mL DNPH (10 mM in HCl 2.0 M) and incubated for 1 h at 37 °C. 500 mL of TCA 20%$_{w/v}$ was added and homogenized, and the resultant mixture was centrifuged at 11,000 RPM, 4 ºC for 5 min. The pellet was washed with EtOH/EtOAc 1:1 (3x100 mL) and resuspended in guanidine (6 M in Phosphate Buffer 20 mM) for 15 min at 37 ºC. Carbonyl concentration was determined the absorbance at 370 nm (e = 22x10^3 L/mol.cm)

and results were expressed in nmol of carbonyl/mg of protein. Experiments were conducted in triplicate as independent assays.

i. Statistical Analysis

All experiments were repeated at least three times in independent replicates and the results of cellular tests were expressed as the mean values ± standard deviations. ANOVA with Bonferroni's test was used to evaluate the differences between cell groups (negative control versus treatment) for all experiments.

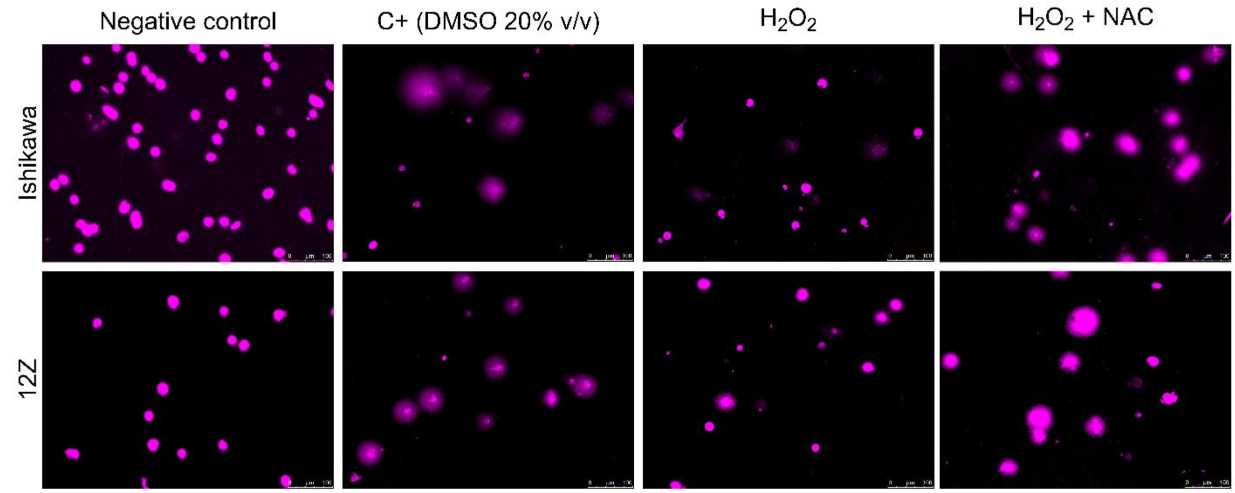

Figure **S1** - **Comet assay (single-cell gel electrophoresis) assessing DNA damage in Ishikawa and 12Z cell lines under different experimental conditions**. Cells were treated with negative control, positive control (C+, 20% v/v DMSO), hydrogen peroxide (H_2O_2), and H_2O_2 combined with N-acetylcysteine (NAC). Representative images show stained DNA (magenta), where increased comet tail intensity and length indicate higher levels of DNA strand breaks. Increased genotoxic damage is observed in cells exposed to H_2O_2 and in the positive control, whereas NAC co-treatment partially attenuates this effect, suggesting antioxidant activity. Scale bars: 100 μm.

Table **S1 - Numerical values correspond to the data presented graphically in the Results section**. The table presents the average values obtained in the experiments, accompanied by their respective standard deviations (when applicable), as represented in the graphs. The data are organized by experimental conditions, according to the order and visual identification adopted in the figures.

Assays	Treatment	Ishikawa cells	12Z cells
H_2O_2 cytotoxicity (EC_{50} ± SD mM)		1,300 ± 148.5	296 ± 35.5
NAC cytotoxicity (Cell Viability % in comparison to Negative Control)	1 mM	105.96 ± 5.73	117.38 ± 15.11
	5 mM	142.69 ± 24.31	184.38 ± 26.23
	10 mM	154.98 ± 20.18	229.67 ± 29.81
NAC Antioxidant Effect (Cell Viability % in comparison to H_2O_2 treated cells)	1 mM	34.80 ± 6.84	171.39 ± 31.86
	5 mM	156.54 ± 29.99	199.82 ± 28.47
	10 mM	135.28 ± 24.26	185.16 ± 43.05
H_2O_2 cytotoxicity - Spheroids (LIVE %)	37 µM	-	28.30
	75 µM	-	28.53
	112.5 µM	28.58	-
	150 µM	-	29.05
	225 µM	28.88	-
	300 µM	-	29.82
	450 µM	27.31	-
	1,300 µM	23.83	-
NAC cytotoxicity - Spheroids (LIVE %)	5 mM	65.80	59.73
	2.5 mM	69.15	60.77
	1.25 mM	66.87	63.31
NAC Antioxidant Effect - Spheroids (LIVE %)	5 mM	67.63	62.32
	2.5 mM	65.46	62.85
	1.25 mM	62.55	65.22
ROS Production - Spheroids (Mean Gray Value)	C-	6.84	25.96
	C+	17.78	37.25
	H_2O_2	20.01	45.81
	H_2O_2 + NAC	11.23	34.14
GSH/GSSG ratio	C-	6.02	4.06
	H_2O_2	4.75	3.1
	H_2O_2 + NAC	5.48	3.28
GSH/GSSG ratio – Spheroids	C-	5.32	3.59
	H_2O_2	4.77	3.76
	H_2O_2 + NAC	4.44	3.52

Total Glutathione (x10^{-7}mol/mg of protein)	C-	2.12	2.69
	H_2O_2	1.77	12.05
	H_2O_2 + NAC	5.46	5.92
Total Glutathione (x10^{-7}mol/mg of protein) – Spheroids	C-	4.73	3.21
	H_2O_2	5.13	0.89
	H_2O_2 + NAC	4.66	1.22
DNA Damage - Comet Assay (Tail Length)	C-	550	670
	C+	750	2.13
	H_2O_2	520	1.13
	H_2O_2 + NAC	1.29	1.09
Lipid Peroxidation - TBARS Assay (nmol of MDA/mg of protein)	C-	330.24 ± 68.17	337.61 ± 81.96
	H_2O_2	619.50 ± 69.06	886.42 ± 230.93
	H_2O_2 + NAC	213.63 ± 49.58	463.71 ± 151.74
Protein Oxidation - DNPH Assay (nmol of carbonyl/mg of protein)	C-	167.45 ± 18.30	414.55 ± 141.40
	H_2O_2	239.23 ± 14.33	1,252.64 ± 369.95
	H_2O_2 + NAC	204.24 ± 11.15	451.87 ± 101.90

References

1. Nunes, E.A.; Manieri, T.M.; Matias, A.C.; Bertuchi, F.R.; Da Silva, D.A.; Lago, L.; Sato, R.H.; Cerchiaro, G. Protective Effects of Neocuproine Copper Chelator against Oxidative Damage in NSC34 Cells. *Mutation Research/Genetic Toxicology and Environmental Mutagenesis* **2018**, *836*, 62–71, doi:10.1016/j.mrgentox.2018.06.019.
2. Wendel, J.R.H.; Wang, X.; Smith, L.J.; Hawkins, S.M. Three-Dimensional Biofabrication Models of Endometriosis and the Endometriotic Microenvironment. *Biomedicines* **2020**, *8*, 525, doi:10.3390/biomedicines8110525.
3. Gomes, K.S.; Coelho, J.A.; Gomes, R.N.; Bosquetti, L.M.; Lange, C.N.; Batista, B.L.; Cerchiaro, G.; Lago, J.H.G. Dehydrodieugenol Isolated from Ocotea Cymbarum Induces Cell Death in Human Breast Cancer Cell Lines by Dysregulation of Intracellular Copper Concentration. *Chemico-Biological Interactions* **2024**, *396*, 111039, doi:10.1016/j.cbi.2024.111039.
4. Afzal, M.; Matsugo, S.; Sasai, M.; Xu, B.; Aoyama, K.; Takeuchi, T. Method to Overcome Photoreaction, a Serious Drawback to the Use of Dichlorofluorescin in Evaluation of Reactive Oxygen Species. *Biochemical and Biophysical Research Communications* **2003**, *304*, 619–624, doi:10.1016/S0006-291X(03)00641-7.
5. Kalyanaraman, B.; Darley-Usmar, V.; Davies, K.J.A.; Dennery, P.A.; Forman, H.J.; Grisham, M.B.; Mann, G.E.; Moore, K.; Roberts, L.J.; Ischiropoulos, H. Measuring Reactive Oxygen and Nitrogen Species with Fluorescent Probes: Challenges and Limitations. *Free Radical Biology and Medicine* **2012**, *52*, 1–6, doi:10.1016/j.freeradbiomed.2011.09.030.
6. Forman, H.J.; Augusto, O.; Brigelius-Flohe, R.; Dennery, P.A.; Kalyanaraman, B.; Ischiropoulos, H.; Mann, G.E.; Radi, R.; Roberts, L.J.; Vina, J.; et al. Even Free Radicals Should Follow Some Rules: A Guide to Free Radical Research Terminology and Methodology. *Free Radical Biology and Medicine* **2015**, *78*, 233–235, doi:10.1016/j.freeradbiomed.2014.10.504.
7. Matias, A.C.; Biazolla, G.; Cerchiaro, G.; Keppler, A.F. α-Aryl-N-Aryl Nitrones: Synthesis and Screening of a New Scaffold for Cellular Protection against an Oxidative Toxic Stimulus. *Bioorganic & Medicinal Chemistry* **2016**, *24*, 232–239, doi:10.1016/j.bmc.2015.12.007.
8. Tietze, F. Enzymic Method for Quantitative Determination of Nanogram Amounts of Total and Oxidized Glutathione: Applications to Mammalian Blood and Other Tissues. *Analytical Biochemistry* **1969**, *27*, 502–522, doi:10.1016/0003-2697(69)90064-5.
9. Abílio, V. Vitamin E Attenuates Reserpine-Induced Oral Dyskinesia and Striatal Oxidized Glutathione/Reduced Glutathione Ratio (GSSG/GSH) Enhancement in Rats. *Progress in Neuro-Psychopharmacology and Biological Psychiatry* **2003**, *27*, 109–114, doi:10.1016/S0278-5846(02)00340-8.
10. Eyer, P.; Podhradský, D. Evaluation of the Micromethod for Determination of Glutathione Using Enzymatic Cycling and Ellman's Reagent. *Analytical Biochemistry* **1986**, *153*, 57–66, doi:10.1016/0003-2697(86)90061-8.
11. [48] Assay of Glutathione, Glutathione Disulfide, and Glutathione Mixed Disulfides in Biological Samples. In *Methods in Enzymology*; Elsevier, 1981; Vol. 77, pp. 373–382 ISBN 978-0-12-181977-4.
12. Toborek, M.; Hennig, B. Fatty Acid–Mediated Effects on the Glutathione Redox Cycle in Cultured Endothelial Cells. *The American Journal of Clinical Nutrition* **1994**, *59*, 60–65, doi:10.1093/ajcn/59.1.60.
13. Bains, J.S.; Shaw, C.A. Neurodegenerative Disorders in Humans: The Role of Glutathione in Oxidative Stress-Mediated Neuronal Death. *Brain Research Reviews* **1997**, *25*, 335–358, doi:10.1016/S0165-0173(97)00045-3.
14. de Sousa, F.S.; Nunes, E.A.; Gomes, K.S.; Cerchiaro, G.; Lago, J.H.G. Genotoxic and Cytotoxic Effects of Neolignans Isolated from Nectandra Leucantha (Lauraceae). *Toxicology in Vitro* **2019**, *55*, 116–123, doi:10.1016/j.tiv.2018.12.011.

15. Bivehed, E.; Hellman, B.; Wenson, L.; Stenerlöw, B.; Söderberg, O.; Heldin, J. Visualizing DNA Single- and Double-Strand Breaks in the Flash Comet Assay by DNA Polymerase-Assisted End-Labelling. *Nucleic Acids Research* **2024**, *52*, e22–e22, doi:10.1093/nar/gkae009.
16. Matias, A.C.; Villa Dos Santos, N.; Chelegão, R.; Nomura, C.S.; Fiorito, P.A.; Cerchiaro, G. Cu(GlyGlyHis) Effects on MCF7 Cells: Copper Uptake, Reactive Oxygen Species Generation and Membrane Topography Changes. *Journal of Inorganic Biochemistry* **2012**, *116*, 172–179, doi:10.1016/j.jinorgbio.2012.07.012.
